# Platinum nanoparticles from size adjusted functional colloidal particles generated by a seeded emulsion polymerization process

**DOI:** 10.3762/bjnano.2.50

**Published:** 2011-08-18

**Authors:** Nicolas Vogel, Ulrich Ziener, Achim Manzke, Alfred Plettl, Paul Ziemann, Johannes Biskupek, Clemens K Weiss, Katharina Landfester

**Affiliations:** 1 Max Planck Institute for Polymer Research, Ackermannweg 10, 55128 Mainz, Germany; 2 Department of Organic Chemistry III, University of Ulm, Albert-Einstein-Allee 11, 89081 Ulm, Germany; 3 Department of Solid State Physics, University of Ulm, Albert-Einstein-Allee 11, 89081 Ulm, Germany; 4 Central Facility of Electron Microscopy, University of Ulm, Albert-Einstein-Allee 11, 89081 Ulm, Germany

**Keywords:** colloid lithography, functional colloids, miniemulsion polymerization, nanoparticles, seeded emulsion polymerization

## Abstract

The benefits of miniemulsion and emulsion polymerization are combined in a seeded emulsion polymerization process with functional seed particles synthesized by miniemulsion polymerization. A systematic study on the influence of different reaction parameters on the reaction pathway is conducted, including variations of the amount of monomer fed, the ratio of initiator to monomer and the choice of surfactant and composition of the continuous phase. Critical parameters affecting the control of the reaction are determined. If carefully controlled, the seeded emulsion polymerization with functional seed particles yields monodisperse particles with adjustable size and functionalities. Size-adjusted platinum-acetylacetonate containing latex particles with identical seed particles and varied shell thicknesses are used to produce arrays of highly ordered platinum nanoparticles with different interparticle distances but identical particle sizes. For that, a self-assembled monolayer of functional colloids is prepared on a solid substrate and subsequently treated by oxygen plasma processing in order to remove the organic constituents. This step, however, leads to a saturated state of a residual mix of materials. In order to determine parameters influencing this saturation state, the type of surfactant, the amount of precursor loading and the size of the colloids are varied. By short annealing at high temperatures platinum nanoparticles are generated from the saturated state particles. Typically, the present fabrication method delivers a maximum interparticle distance of about 260 nm for well-defined crystalline platinum nanoparticles limited by deformation processes due to softening of the organic material during the plasma applications.

## Introduction

Uniform colloidal particles have attracted attention from various research fields for their ability to crystallize in highly symmetric arrangements. Two-dimensional crystals, commonly referred to as colloidal monolayers, are widely used for lithographic processes to create metal nanostructures in a cheap and highly parallel fashion [1]. As it is not a light-based process, the diffraction limit is conveniently circumvented and nanostructures with dimensions of only several tens of nanometers are created with remarkable ease. While the conventional process, leading to triangular shaped particle arrays, has long been established [2–3], research is focused on the creation of more sophisticated structures [4], including embedded objects [5–6], rings [7], discs [8] and crescent shaped particles [9–10].

While the majority of work on lithographic applications deals with plain colloidal particles, the incorporation of functionalities leads to different structural designs. In particular, the incorporation of metal complexes into polymer particles assembled into 2D crystals has recently been used as a non-conventional lithography approach to construct highly symmetrical arrays of metal nanoparticles (NPs) with dimensions of only several nanometers [11–12]. In contrast to conventional colloidal lithography, this approach employs the functional colloids as sacrificial carriers, rather than, e.g., being used as masks for metal evaporation. The size of the resulting metal NPs is determined by the quantity of complex in the precursor loaded colloids. Their adjustable size defines the interparticle distance of the NPs, and simultaneous loading with two metal complexes gives access to the fabrication of alloy NPs.

A number of different synthetic approaches for colloidal particles is known in literature, most prominently emulsion and miniemulsion polymerization. Although both yield polymeric colloidal particles, they differ both in reaction mechanism as well as in the properties of the resulting particles. Surfactant-free emulsion polymerization is a diffusion controlled process that is praised for excellent monodispersity and precise control of the particle size [13–15]. However, problems arise when the incorporation of functionalities (e.g., co-monomers, dyes, metal complexes) is required, as the different diffusion coefficients of monomer and functional molecule complicate the incorporation. Quantitative incorporation, for example, to create a precise stoichiometry of several different molecules within a latex particle, is thus impeded by emulsion polymerization [16].

Miniemulsion polymerization on the contrary is a powerful tool for the synthesis of highly functional polymeric nanoparticles [17–20]. Here, the monomer droplets are preformed by ultrasonication and critically stabilized against coagulation by the addition of surfactants. Ostwald ripening, the mechanism that leads to formation of bigger particles at the expense of smaller ones due to the higher Laplace pressure of the latter, is prevented by addition of a co-stabilizer. This component, highly insoluble in the continuous phase, creates an osmotic pressure in the droplets and, thus, acts as a counterforce to the Laplace pressure. Hence, no effective diffusion takes place during the polymerization, and functional molecules can be incorporated in defined amounts. The only requirement for the incorporation is a higher solubility of the functional molecule in the monomer droplets as compared to the continuous phase. As many different monomers can be used, and both direct (oil-in-water) and indirect (water-in-oil) processes are accessible, solubility is not a significant limitation for the majority of molecules. In recent years, the incorporation of functionalities into polymeric particles was thoroughly explored and includes fluorescent molecules [21], metal complexes [16], pigments [22], quantum dots [11] and magnetic particles [23].

In this article, we report on studies undertaken to determine process parameters for the creation of advanced colloidal monolayer architectures. A seeded emulsion polymerization process was applied and used to combine the benefits of both emulsion and miniemulsion polymerization. Using a miniemulsion polymerization process, functional seed particles were synthesized. Subsequent application of an emulsion-like polymerization in the presence of seed particles allowed the control of the size and polydispersity of such functional latex particles. Subsequently, these particles could then be crystallized into functional 2-D or 3-D colloidal crystals. As an application, we demonstrate, in subsection 2 of the Results and Discussion, the use of platinum containing spheres in an etching process leading to arrays of platinum NPs with controlled interparticle distances [11–12].

## Results and Discussion

### 1 Adjustment of colloidal size by a seeded emulsion polymerization process

**Scheme 1:**
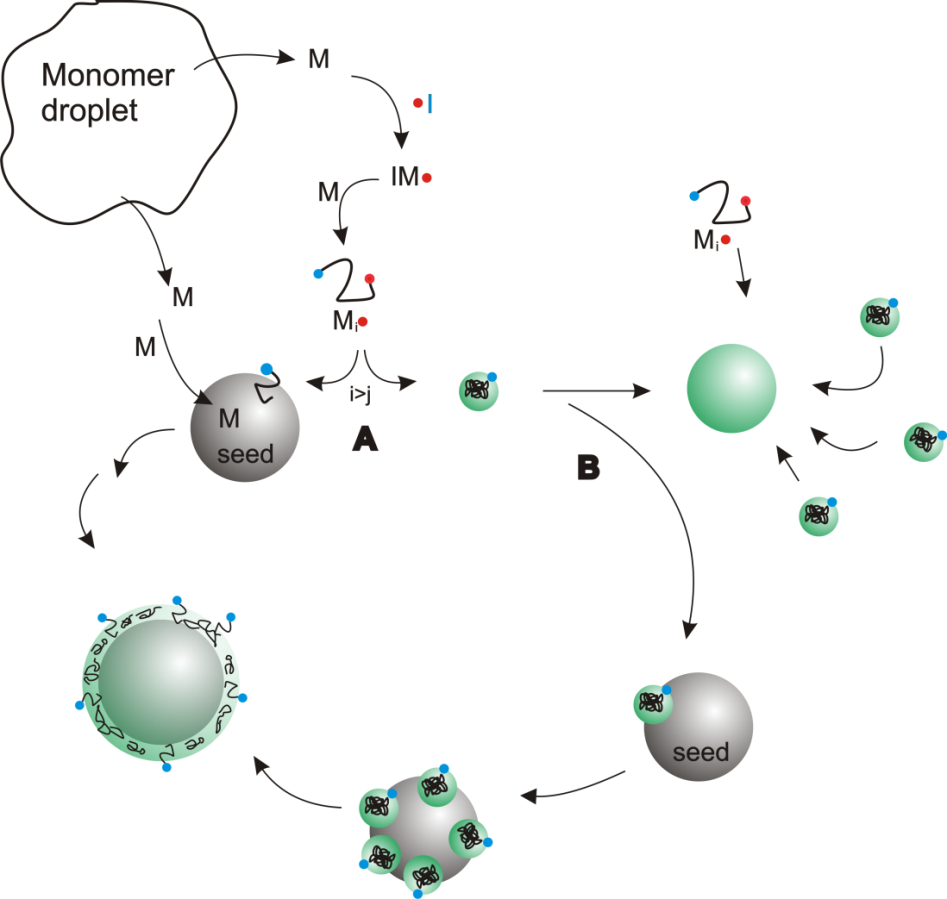
Simplified model of a seeded emulsion polymerization process to clarify the desired reaction pathway.

**Figure 1:**
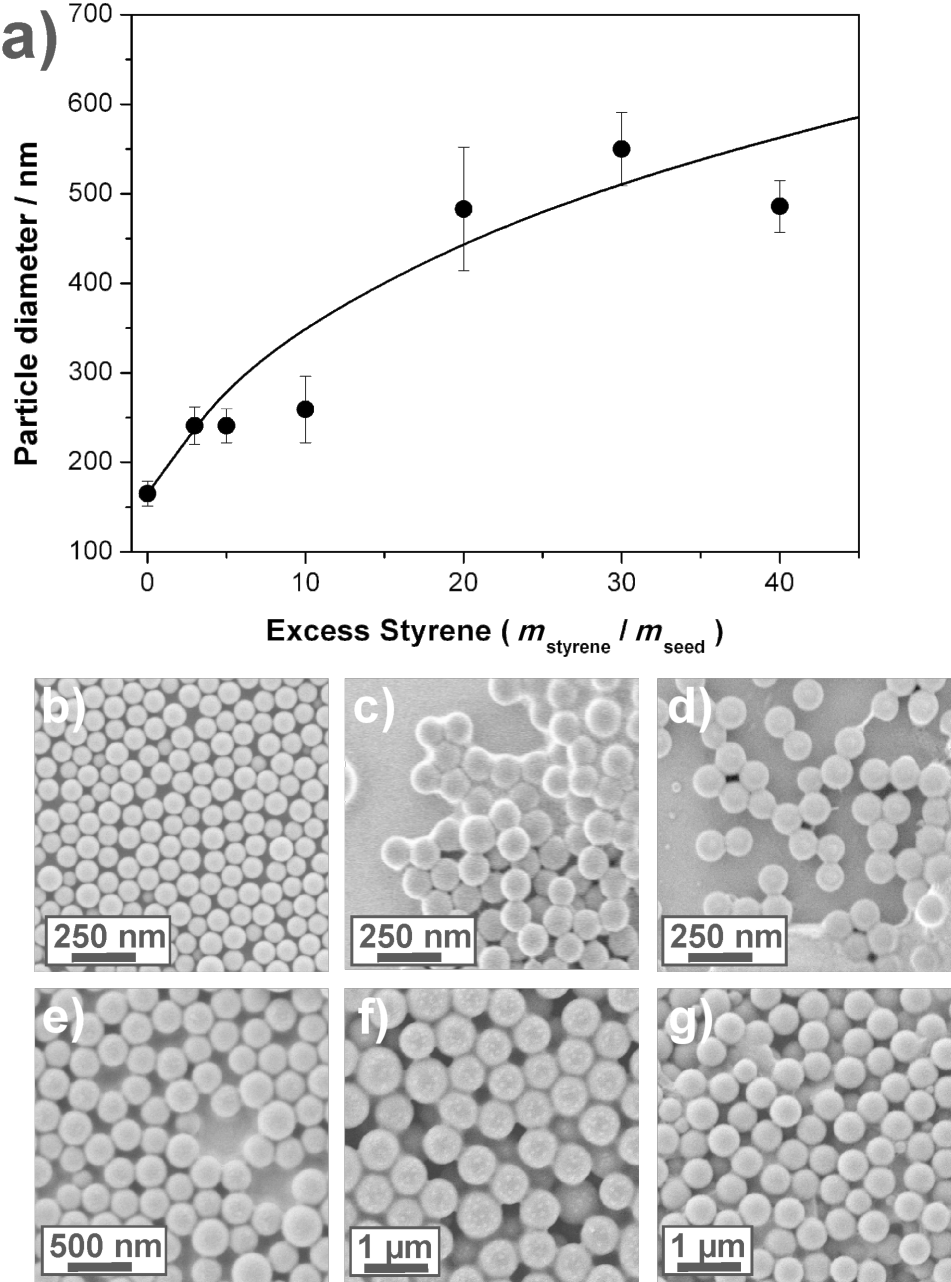
Variation of the amount of monomer added to control the resulting size in the seeded emulsion polymerization process. a) Styrene excess versus size of the resulting particles. The solid curve in the diagram indicates the theoretical expectation, assuming 100% conversion of the excess monomer added (diameter ~ (excess)^1/3^).; b–g) SEM images of the resulting particles: b) seed particles as produced by a miniemulsion process; c) 3-fold excess of styrene; d) 5-fold excess; e) 10-fold excess; f) 30-fold excess; g) 40-fold excess.

In order to combine the advantages of both miniemulsion- and emulsion polymerization to create monodisperse functional colloidal particles with precisely adjustable sizes, a seeded emulsion polymerization process was adopted and the influence of principal reaction parameters on the resulting particles was investigated. Scheme 1 shows the mechanism of the seeded emulsion polymerization process. Latex particles loaded with platinum acetylacetonate as a model compound for a functional molecule, were prepared by a conventional miniemulsion polymerization process [24]. The reaction parameters are specified in the Experimental section. The process resulted in polystyrene latex particles with a size of 167 ± 14 nm and a platinum acetylacetonate load of 1 wt %. They were used as seed particles for the investigations that are presented in the following. A representative scanning electron microscopy (SEM) image of the particles is shown in Figure 1b (see below).

All seeded emulsion polymerization reactions were executed as follows. A dispersion of 0.1 wt % of seed particles with 0.01 wt % of sodium dodecylsulfate (SDS, relative to the amount of water used) was heated to 75 °C. Ammonium peroxodisulfate, (APS, (NH_4_)_2_S_2_O_8_), used as initiator, was dissolved in a small amount of ultra-pure water and added to the dispersion. Styrene, as monomer, was added to the solution using a syringe pump with a flow rate of 1 mL·h^−1^. The solution was stirred for 24 h at 80 °C under an argon atmosphere. After completion, the resulting particles were dialyzed in order to remove the unreacted initiator and monomer. Previously, it has been shown by inductively coupled plasma optical emission spectrometry (ICP-OES) that a seeded emulsion polymerization process does not change the amount of platinum acetylacetonate in the particle [24].

### 1.1 Polymerization mechanism and factors influencing secondary nucleation

For the detailed investigation of the influences of the different reaction parameters, it is essential to examine the reaction mechanism in detail. The focus of the present study lies in tuning the reaction in such a way as to avoid secondary nucleation. Secondary nucleated particles are colloids newly formed in the course of the reaction, similar to the formation of latex particles in a conventional emulsion polymerization [25–26]. Given that the seeded emulsion polymerization is applied to adjust the size of the final functional colloidal particles, secondary nucleation must be prevented in order to guarantee that all particles bear the desired functionality.

Scheme 1 gives a simplified picture of the processes involved. After injection, the monomer diffuses through the water phase to the hydrophobic seed particles, which thus represent a monomer rich area. The initiator thermally decomposes to form radicals in the water phase. These start to polymerize monomer molecules that are present in the aqueous phase, to form oligoradicals IM_i_·. Due to the ionic head group introduced by the persulfate radical, the oligoradicals remain water soluble until they reach a critical chain length IM_j_·, upon which they become insoluble in water [26–27]. For styrene, this length was found to be five monomer units [27–28]. The fate of the water soluble oligoradical and, thus, the development of the reaction, is determined by the question of whether or not it enters a seed particle before reaching the critical chain length. This point is marked as A in Scheme 1. Entering a seed particle gives the radical access to the monomer reservoir present in the particle, where it subsequently polymerizes, resulting in a size-increased seed particle (Scheme 1, left side). Assuming the oligoradical does not meet a seed particle before adding the last monomer unit necessary to exceed the critical chain length, the chain becomes insoluble in the water phase and forms a particle nucleus by a coil-to-globule transition (this pathway of the reaction is shown on the right side of the scheme) [25,27,29]. This particle nucleus itself is not stable as it features only one charge from the initiator. Stabilization can be achieved by two different pathways, marked with a B in Scheme 1. First, the particle nucleus can attach to a seed particle, where it will eventually be completely incorporated over the course of reaction due to more diffusion of monomers to the particle. In this case, no secondary particle will appear in the final dispersion [25–26]. On the other hand, several particle nuclei can cluster together to form a stable particle when the charge density on their surface becomes sufficiently high [25,27,29]. This particle, termed the secondary particle (shown in green color) now participates in the reaction as a new seed particle. In order to avoid secondary nucleation forming unwanted, plain particles without a metal precursor, the latter pathway needs to be avoided. Several factors influence the course of the reaction: Predominantly the seed concentration and the concentration of surfactant added to the reaction. Furthermore, the initiator concentration and the composition of the continuous phase important for determining the fate of the radicals as well as of the particle nuclei. Various studies have been published, especially on the role of seed particle concentration [30–31]. It was found that concentrations above 10^14^ particles per litre are sufficient to avoid the formation of particle nuclei [30]. Therefore, for all reactions performed, the seed particle concentration was fixed to 2.65 × 10^14^ particles per litre and the effect of the other reaction parameters was investigated.

Furthermore, in all cases styrene was added dropwise, very slowly at a rate of 1 mL·h^−1^. This ensured that the monomer was directly consumed once it appeared in the reaction mixtures (‘monomer starved condition’) [32] and no monomer reservoir was present that might steer the reaction pathway towards secondary nucleation in a classical emulsion polymerization way.

Seed particles containing platinum-acetylacetonate were used in all experiments in order to establish a procedure to tune, systematically, the size of the functional colloidal particles. Metal-complex containing polymer latexes are promising materials for a nonconventional lithography approach to produce ordered arrays of platinum nanoparticles [11]. Here, it is of particular importance that no secondary particles are generated, as otherwise, in the finally obtained array of Pt nanoparticles, some of them would be randomly missing, hence deteriorating the desired order.

### 1.2 Variation of the amount of monomer added

In a first set of experiments, the amount of monomer added was varied while all other parameters remained constant. These experiments were aimed at the investigation of the available size range for the seeded emulsion polymerization. The amount of monomer added is described as the normalized quantity *monomer excess* and describes the mass of styrene added relative to the total mass of seed particles (*m*_styrene_/*m*_seed_). Please note that the total amount of initiator was unchanged, leading to a constant initiator concentration in the water phase, but drastically changing the ratio of initiator to monomer added. Table 1 presents the reaction details, Figure 1 shows the dependency of the amount of monomer added on the resulting particle size.

**Table 1:** Reaction details for the set of experiments shown in Figure 1.

styrene added		H_2_O	*N*_seed_ (wt %)	[I]	[I]/[M] ratio	[I]/[H_2_O] ratio	SDS conc.	diameter SEM (theory)
*m*_styrene_/*m*_seed_	/mg	/g	/L^−1^	/mg	/%	/%	/wt % of H_2_O	/nm

3	150	50	2.65 × 10^14^ (0.1)	250	166.7	0.5	0.01 (5 mg)	241 ± 21 (238)
5	250	50	2.65 × 10^14^ (0.1)	250	100.0	0.5	0.01 (5 mg)	241 ± 19 (282)
10	500	50	2.65 × 10^14^ (0.1)	250	50.0	0.5	0.01 (5 mg)	255 ± 37 (355)
20	1000	50	2.65 × 10^14^ (0.1)	250	25.0	0.5	0.01 (5 mg)	483 ± 68 (448)
30	1500	50	2.65 × 10^14^ (0.1)	250	16.7	0.5	0.01 (5 mg)	550 ± 41 (513)
40	2000	50	2.65 × 10^14^ (0.1)	250	12.5	0.5	0.01 (5 mg)	488 ± 28^a^ (564)
50	2500	50	2.65 × 10^14^ (0.1)	250	10.0	0.5	0.01 (5 mg)	multimodal (608)
100	5000	50	2.65 × 10^14^ (0.1)	250	5.0	0.5	0.01 (5 mg)	multimodal (766)

^a^appearance of secondary nucleated particles.

The solid curve in panel a) of Figure 1 indicates the theoretically expected size of the colloids assuming full conversion of the monomer in the seed particles, leading to the relation: Diameter ~ (excess)^1/3^. The experimentally found colloidal diameters follow the relation reasonably well up to a monomer excess of approximately 30. For higher amounts (<50-fold excess) of monomer added, secondary particles appear leading to a reduction in size of the primary, seeded particles. Even higher monomer amounts (≥50-fold excess) lead to multimodal size distributions indicating uncontrolled reactions with secondary nucleation as the primary reaction pathway [30]. SEM images of the differently produced particles are shown in Figure 1b–g. Compared to the seed particles (Figure 1b), the size enhanced particles exhibit a higher homogeneity. From the present set of experiments one concludes that particles with diameters up to at least 500 nm can be synthesized in a single reaction, a number that relates to an almost 40-fold increase in volume. Larger diameters of seeded colloids could not be achieved as the present reaction pathway appears to break down with higher monomer excesses: Secondary nucleation or instable dispersions are the result.

As all reactions produced an increase of particle size, it can be stated that the initiator concentration in the aqueous phase was high enough to provide enough radicals for a successful polymerization. However, with the initiator concentration being constant in the aqueous phase, we face the unfavourable situation of having an enormous amount of initiator relative to the amount of monomer added for small monomer excesses. Hence, the degree of polymerization is low, and oligomers are formed that reside in the aqueous phase. These oligomers are seen in the SEM images in Figure 1c and Figure 1d as an undefined film covering the colloidal particles, appearing as bright seams. In the following section, the influence of the initiator concentration is discussed in more detail. In general, the molecular weight of the polymer is of minor importance for colloidal synthesis as long as the degree of polymerization is high enough to ensure that the polymeric chains will be found inside the particle.

### 1.3 Variation of the initiator to monomer ratio [I]/[M]

**Figure 2:**
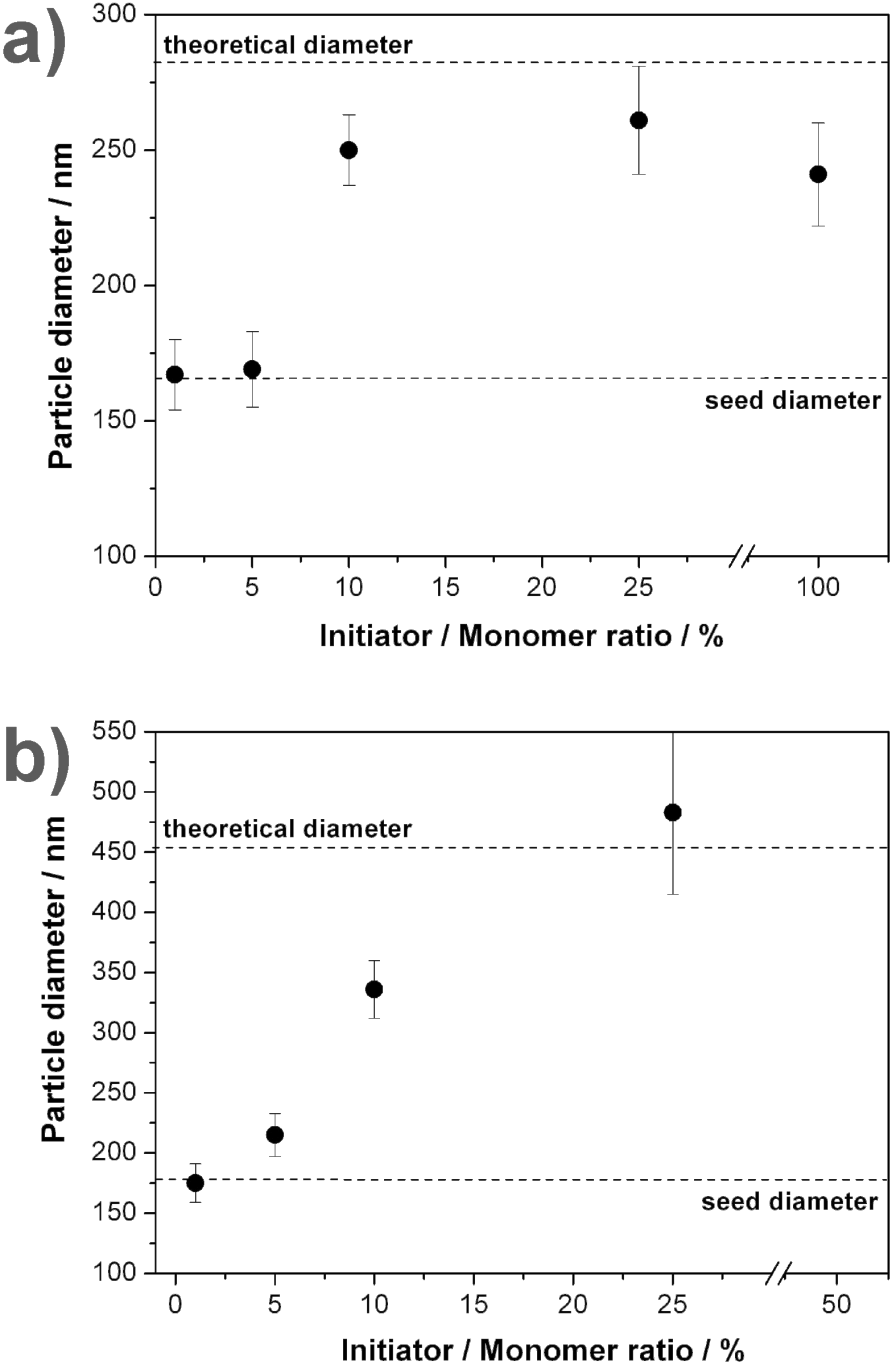
Variation of the amount of initiator relative to the amount of added monomer. The dotted lines are guides to the eye and represent the diameter of the seed particles used in the reaction as well as the theoretical diameter reached for 100% conversion of the additional monomer. a) Reactions with 5-fold excess of styrene with respect to the mass of seed particles. b) Reactions with 20-fold excess of styrene with respect to the mass of seed particles.

Next, the influence of the initiator concentration on the course of the reaction was investigated. In contrast to normal bulk polymerizations, not only is the amount of initiator relative to the monomer important, but also the concentration in the water phase has to be taken into account, as initiation and the first propagation steps take place in the continuous phase. Therefore, the amount of initiator needed is generally higher compared to bulk polymerizations. Figure 2 presents the effect of the initiator concentration on the resulting particle sizes for styrene excesses of 5 (Figure 2a) and 20 (Figure 2b), respectively. The parameters used for the reactions are summarized in Table 2. Dotted lines are inserted into the diagrams to indicate the original size of the seed particles as well as the maximum theoretical size. Both sets of reactions show that lower initiator amounts (1–5% relative to the monomer added) do not induce an increase in size, as the concentration of initiator in the water phase is insufficient to induce polymerization processes. For the optimum initiator concentration for the seeded reactions, as determined for 5-fold and 20-fold monomer excess, we estimate values between 10 and 25 wt % relative to the monomer. At higher quantities of initiator, the resulting particle sizes decrease (cf. Figure 2a) as a result of insufficient polymerization leading to oligomers that remain in the water phase, as seen by the films covering the particles in the SEM micrographs. As shown in previous experiments, the most suitable values for the initiator concentration are not exclusively determined by the amount of added monomer but also by the concentration of radicals in the water phase. Hence, it can be expected that for higher monomer excesses, smaller values for the initiator to monomer ratio will be sufficient to induce polymerization.

**Table 2:** Reaction details for the set of experiments shown in Figure 2.

styrene added		H_2_O	*N*_seed_ (wt %)	[I]	[I]/[M] ratio	[I]/[H_2_O] ratio	SDS conc.	diameter SEM (theory)
*m*_styrene_/*m*_seed_	/mg	/g	/L^−1^	/mg	/%	/%	/wt % of H_2_O	/nm

5	250	50	2.65 × 10^14^ (0.1)	2.5	1	0.005	0.01 (5 mg)	167 ± 13 (282)
5	250	50	2.65 × 10^14^ (0.1)	12.5	5	0.025	0.01 (5 mg)	169 ± 14 (282)
5	250	50	2.65 × 10^14^ (0.1)	25	10	0.05	0.01 (5 mg)	250 ± 14 (282)
5	250	50	2.65 × 10^14^ (0.1)	62.5	25	0.125	0.01 (5 mg)	261 ± 20 (282)
5	250	50	2.65 × 10^14^ (0.1)	250	100	0.5	0.01 (5 mg)	241 ± 19 (282)

20	1000	50	2.65 × 10^14^ (0.1)	10	1	0.02	0.01 (5 mg)	175 ± 16 (448)
20	1000	50	2.65 × 10^14^ (0.1)	50	5	0.1	0.01 (5 mg)	215 ± 18 (448)
20	1000	50	2.65 × 10^14^ (0.1)	100	10	0.2	0.01 (5 mg)	336 ± 24 (448)
20	1000	50	2.65 × 10^14^ (0.1)	250	25	0.5	0.01 (5 mg)	483 ± 68 (448)

### 1.4 Variation of surfactant type and concentration

**Figure 3:**
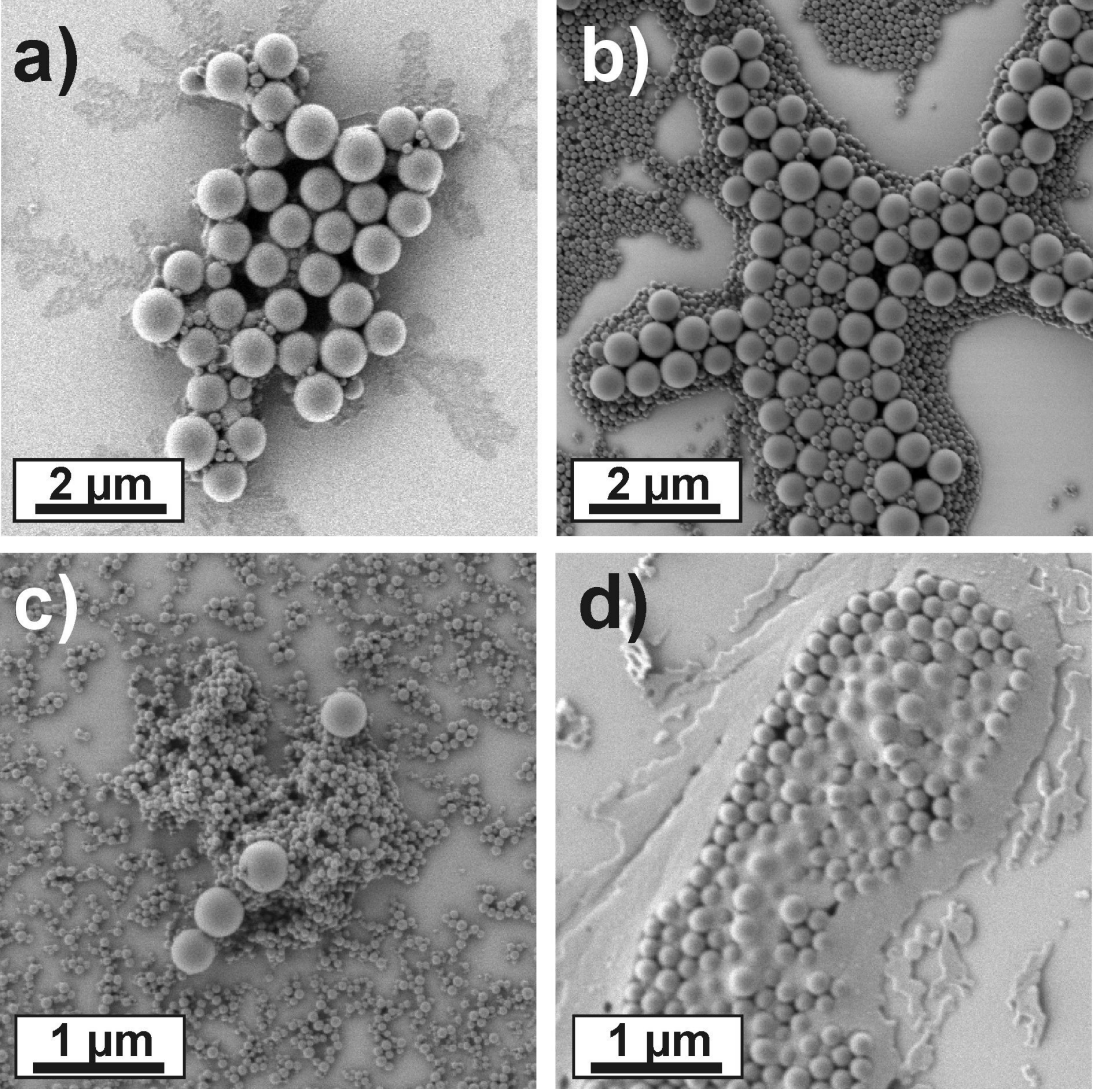
Effect of different concentrations and types of surfactants applied to stabilize the particles in the process. All concentrations given are relative to the weight of the water phase. a) 0.01 wt % SDS. b) 0.05 wt % SDS. c) 0.1 wt % SDS. d) 0.1 wt % Lutensol AT50.

It is to be expected that the type and amount of surfactant added to stabilize the seeded particles should have a significant influence on the reaction pathway [33]. This becomes clear when considering that the surfactant not only stabilizes the growing seed particles but can also adsorb to and stabilize undesirable nucleated secondary particles. Thus, it is expected that the amount of surfactant should be minimized to that concentration necessary to yield a stable emulsion. Figure 3 and Table 3 present the experimental data for reactions performed with varying surfactant type and concentrations. As a test system, the reaction with a styrene excess of 100 was chosen as this led to multimodal size distributions in previous experiments (Figure 3a shows the result of the standard reaction). Obviously, the amount of SDS added in the standard recipe is insufficient to induce stable reaction conditions. Hence, the SDS concentration in the continuous phase was increased from 0.01 wt % up to 0.1 wt %. All SDS concentrations were below the critical value for micelle formation (cmc). Such concentrations are used in conventional emulsion polymerization and lead to particles nucleated in monomer swollen micelles [13]. Figure 3b and c show representative SEM micrographs of the resulting dispersions. For both cases, massive secondary nucleation took place, leading to bimodal size distributions. In the first case (0.05 wt % SDS), the size enhanced seed particles feature an excellent monodispersity and have a size of approximately 600 nm, indicating a more stable course of reaction. In contrast, higher amounts of SDS stabilize the particle nuclei more efficiently and induce massive secondary nucleation. With a SDS concentration of 0.1 wt %, almost all of the monomer added is converted into secondary particles, thus resembling a conventional emulsion polymerization. Only a minor fraction of size enhanced seed particles is found (Figure 3c). Changing the type of surfactant from the anionic SDS to the non-ionic Lutensol AT50 compromised the reaction stability. As depicted in Figure 3d, the reaction did not lead to a successful conversion of the monomer to size enhanced particles. Instead, the seed particles only marginally grew in size, and most of the monomer added was found as a polymeric film covering the complete surface. This is not surprising as non-ionic surfactants are known to stabilize colloids less efficiently than charged ones. Thus, significantly larger amounts of non-ionic surfactants are usually needed to stabilize droplets or particles.

**Table 3:** Reaction details for the set of experiments shown in Figure 3.

styrene added		H_2_O	*N*_seed_ (wt %)	[I]	surfactant	surfactant conc.
*m*_styrene_/*m*_seed_	/mg	/g	/L^−1^	/mg		/wt % of H_2_O

100	250	50	2.65 × 10^14^ (0.1)	250	SDS	0.01 (5 mg)
100	250	50	2.65 × 10^14^ (0.1)	250	SDS	0.05 (25 mg)
100	250	50	2.65 × 10^14^ (0.1)	250	SDS	0.1 (50 mg)
100	250	50	2.65 × 10^14^ (0.1)	250	Lutensol AT50	0.1 (50 mg)

Summarizing these experiments, it can be stated that the amount and type of surfactant crucially influences the reaction pathway. Surfactant has to be added in order to stabilize the growing particles. However, the concentration should be as small as possible. Otherwise, secondary nucleation becomes the primary reaction pathway. As a consequence, the direct conversion of small seed particles to very large ones, above approximately 600 nm, was impeded. From a technological point of view, seeded particles with larger particles have to be synthesized in a step-wise manner, using consecutive seeding reactions. The secondary particles appearing from the reaction with 0.05 wt % SDS could be also successfully removed by centrifugation.

### 1.5 Variation of continuous phase composition

**Figure 4:**
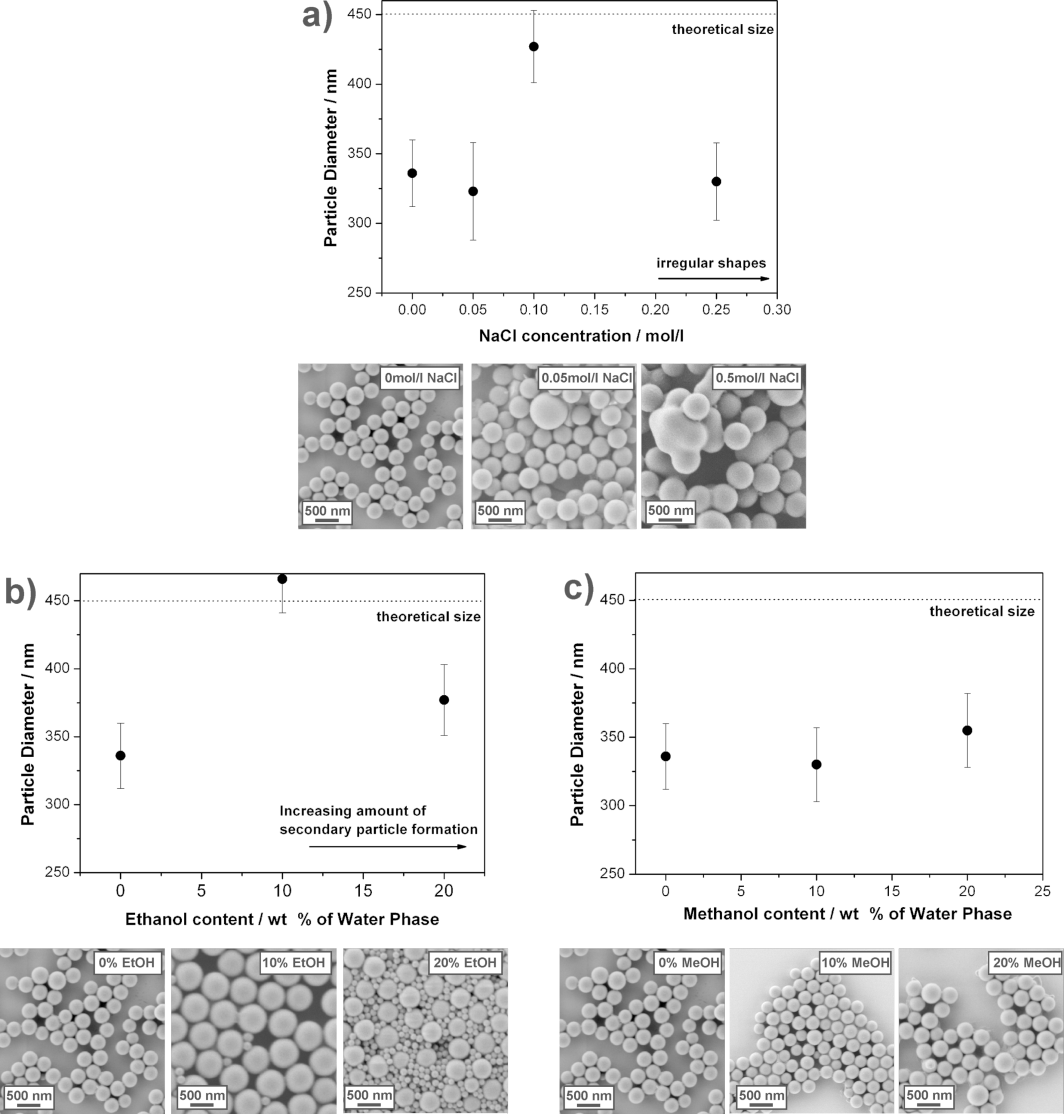
Effects of the composition of the continuous phase on the seeded polymerization reactions. The dotted lines in the diagrams represent the theoretical size of the colloids assuming full conversion of the added monomer. Representative SEM images are shown below the different diagrams. a) Addition of sodium chloride to the continuous water phase. b) Addition of ethanol to the water phase. c) Addition of methanol to the continuous water phase.

The continuous phase has an important influence on the reaction pathway as well. The monomer added has to diffuse through the continuous phase in order to reach the seed particles. Polymerization initiation takes place in the continuous phase and its properties are important for both stabilization of seed particles and nucleation of secondary particles over the course of the reaction. Additionally, both diffusion and solubility of the monomer are affected drastically by compositional changes in the continuous phase. In order to investigate these effects, systematic variations of the continuous phase were performed. Figure 4 and Table 4 show the properties of the resulting dispersions and the reaction details, respectively.

**Table 4:** Reaction details for the set of experiments shown in Figure 4.

styrene added		H_2_O	*N*_seed_ (wt %)	[I]	SDS conc.	addition cont. phase	diameter SEM (theory)
*m*_styrene_/*m*_seed_	/mg	/g	/L^−1^	/mg	/wt % of H_2_O		/nm

20	1000	50	2.65 × 10^14^ (0.1)	100	0.01 (5 mg)	—	336 ± 24 (448)
20	1000	50	2.65 × 10^14^ (0.1)	100	0.01 (5 mg)	60 mg NaCl (0.05 mol·L^−1^)	323 ± 35(448)
20	1000	50	2.65 × 10^14^ (0.1)	100	0.01 (5 mg)	120 mg NaCl (0.1 mol·L^−1^)	427 ± 26 (448)
20	1000	50	2.65 × 10^14^ (0.1)	100	0.01 (5 mg)	300 mg NaCl (0.5 mol·L^−1^)	330 ± 28 (448)
20	1000	50	2.65 × 10^14^ (0.1)	100	0.01 (5 mg)	600 mg NaCl (0.5 mol·L^−1^)	irreg. shapes (448)

20	1000	45	2.65 × 10^14^ (0.1)	100	0.01 (5 mg)	5 mg EtOH (10 wt %)	463 ± 25^a^ (448)
20	1000	40	2.65 × 10^14^ (0.1)	100	0.01 (5 mg)	10 mg EtOH (20%)	377 ± 26^a^ (448)

20	1000	45	2.65 × 10^14^ (0.1)	100	0.01 (5 mg)	5 mg MeOH (10%)	330 ± 27 (448)
20	1000	40	2.65 × 10^14^ (0.1)	100	0.01 (5 mg)	10 mg MeOH (20%)	355 ± 27 (448)

^a^secondary particle formation observed.

First, sodium chloride was added to the continuous phase. The presence of salt affects the stability of colloidal particles as the extension of the electrical double layer of ionic surfactants present at the colloid interface is reduced by the counter ions of the salt. Naturally, this is undesirable for the seed particles. However, it may be that the stability of newly formed secondary particles is reduced as well. Assuming that a secondary particle is composed of a certain number of collapsed chains in order to be stable, the corresponding coagulation of such chains may be suppressed by the addition of small amounts of salt and, thus, the concentration of secondary particles may be strongly reduced without compromising the stability of the seed particles. The experimental data (Figure 4a) seem to support this idea. While very small amounts of sodium chloride (0.05 wt %) did not affect the polymerization, the addition of 0.1 wt % of NaCl induced an increase in final particle size almost to the theoretical value. NaCl concentrations beyond this value interfered with electrostatic stabilization and led to irregularly shaped particles and partial aggregation.

Next, ethanol was added to the continuous phase. In that case, the stability of the latex particles was not crucially affected as only a maximum of 20 wt % of ethanol was added. It was expected that ethanol addition would increase the solubility of styrene in the continuous phase. Moreover, the oligoradical formed would have a different solubility in ethanol-containing water as well, leading to an increase of the critical chain length IM_j_·.

The experimental results, shown in Figure 3b show the following characteristics. First, small amounts of ethanol (10 wt %) induce well-growing seed particles that feature a final size close to the theoretical expected value. A minor amount of secondary particles is visible. Increasing the ethanol content induces a drop in the final diameter close to the value measured for particles synthesized in pure water. Additionally, the amount of secondary particles drastically increases. It is worth mentioning that the particles from reactions with ethanol generally feature a higher monodispersity than the particles prepared in pure water.

Finally, methanol was added to the continuous phase as well (Figure 3c), however, no influence on the reaction was detected, even for a methanol content of 20 wt %. Furthermore, all obtained dispersions closely resembled each other with respect to final size and homogeneity. This may be attributed to the fact that methanol does not act as a solvent for polystyrene.

### 2 Generation of platinum nanoparticles

Colloidal spheres, as prepared by the above described seeding process, were applied for the fabrication of well-ordered Pt NPs of controlled size and interparticle distance on solid substrates. Since the starting size of the colloidal spheres determines the finally obtained interparticle distance of the Pt NPs, the presently introduced novel seeding procedure to tailor that size plays an essential role.

**Figure 5:**
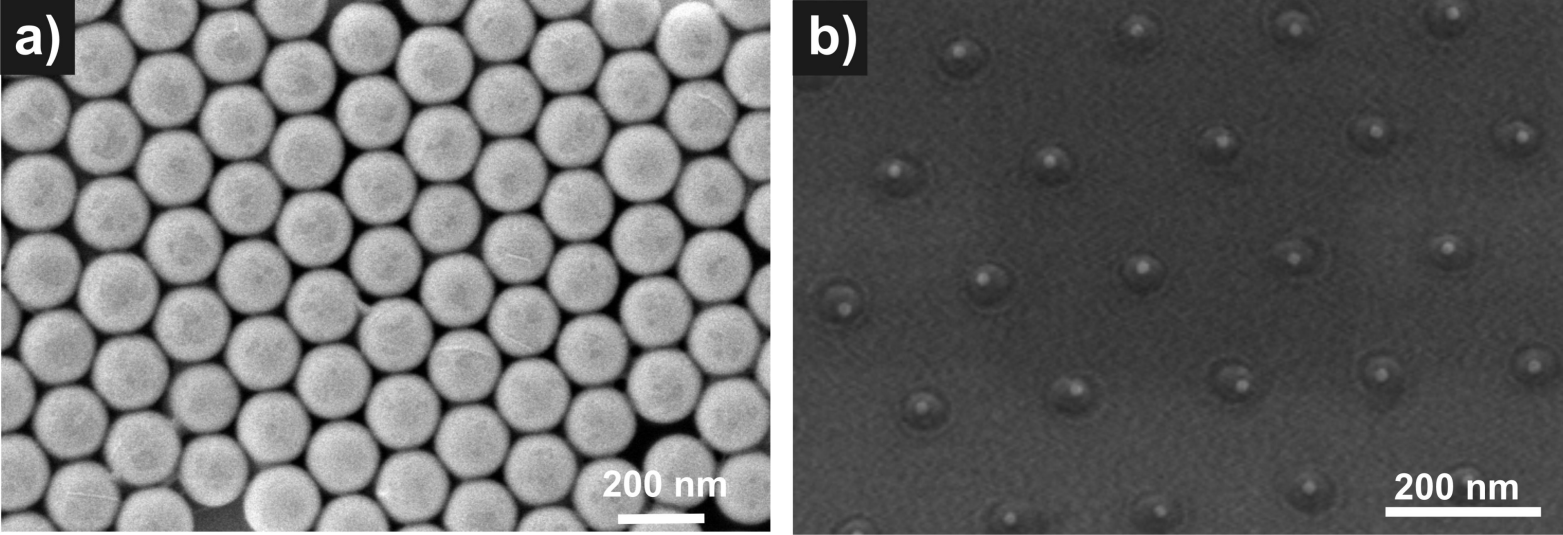
A non-conventional lithographic process is used to produce arrays of Pt NPs from platinum-acetylacetonate containing polymer particles on top of a Si wafer. a) Colloidal monolayer formed by drop casting of seeded colloid particles. b) Arrays of Pt NPs produced by plasma-assisted removal of the organic material and subsequent thermal annealing.

The starting situation and the final state of this fabrication process on a silicon wafer are demonstrated in Figure 5. After depositing a single layer of the precursor loaded colloids on top of Si substrates, their size is reduced by exposure to an isotropic oxygen plasma (see Experimental section). In this way, one arrives at a continuous and laterally homogeneous reduction of the particle diameters while the original particle positions are strictly preserved. The reduction rate of this etching process, imposed by the isotropic plasma, monotonically decreases and finally becomes zero. The corresponding saturation diameters are, however, much larger than what is implied for a pure Pt particle from the amount of metal present within a colloidal sphere. An additional subsequent short annealing in oxygen at 1100 °C (see Experimental section) and cooling down in nitrogen gas-flow is needed to finally obtain well-defined crystalline Pt NPs.

### 2.1 Oxygen plasma exposure: Dependency of the saturation state on different parameters

**Figure 6:**
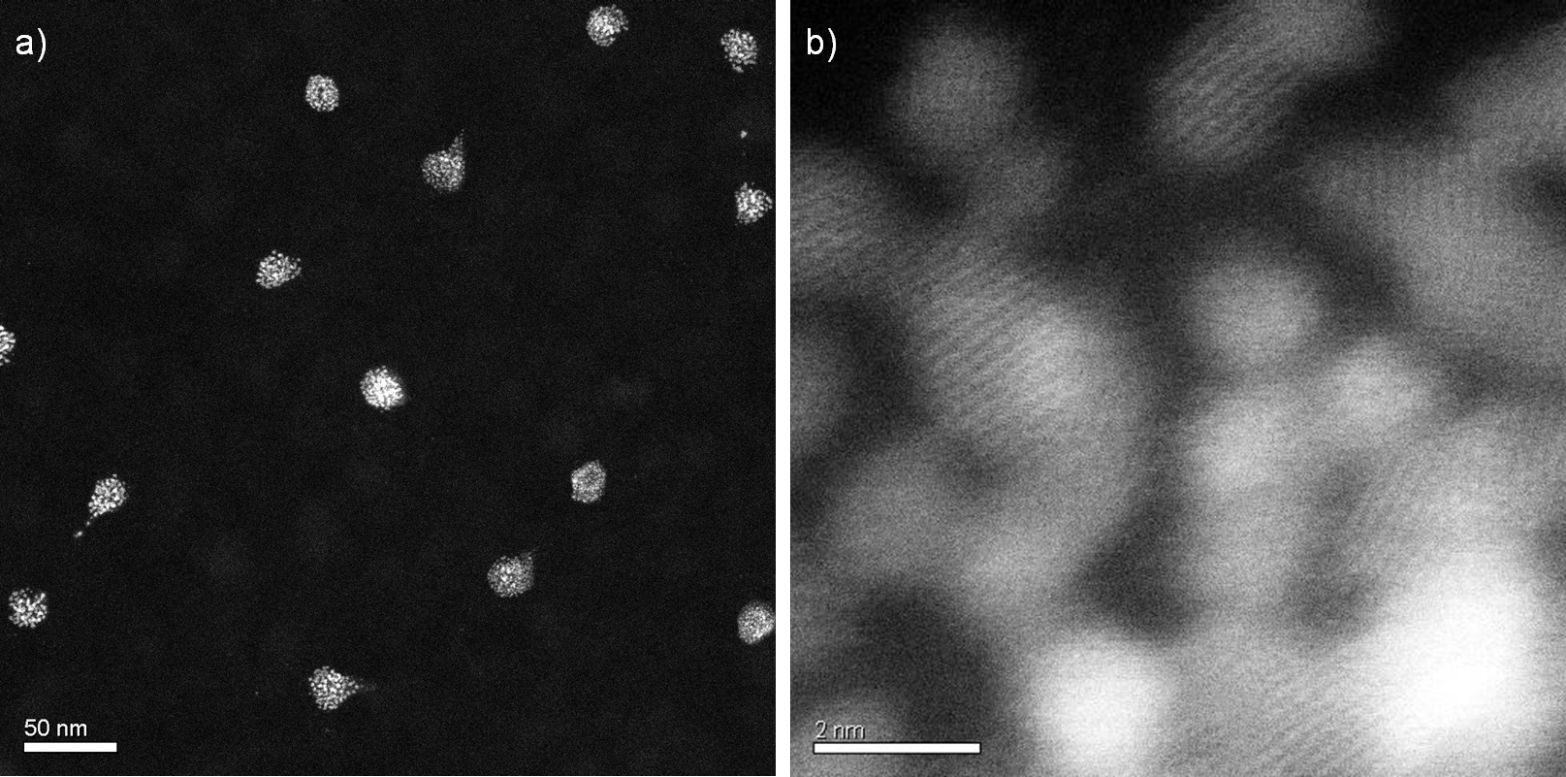
Pt-precursor loaded PS colloids on a Si_3_N_4_ membrane in the saturated state after exposure to isotropic oxygen plasma for 25 min. a) The diameters of the lumps average out to 35 nm. b) Magnified HRSTEM image of one of the particles demonstrates that they consist of an agglomeration of ca. 1–2 nm Pt-rich crystallites.

The plasma procedure was optimized and then performed by default according to the recipe described in the Experimental part. Varying the process parameters is possible within certain limits without influencing the results given below. In the saturated state as obtained by plasma etching, the small lumps forming the hexagonal arrangements always consist of independently nucleated small crystalline particles, which are still embedded in a residual matrix (see the high resolution scanning transmission electron microscope (HRSTEM) images in Figure 6). Some of the particles are not spherical, because they were tilted towards one side during plasma treatment [34]. As proven by X-ray photoelectron spectroscopy (XPS), the lumps always contain PtO_2_. In this state also sodium and sulfur was detected when SDS was used as surfactant. Similar observations for Pt NPs produced from precursor-loaded colloids, prepared with emulsion or miniemulsion polymerization, have already been reported but were not investigated in detail at the time [11–12]. In order to determine the parameters influencing the saturation state, we varied the type of surfactant, the amount of precursor loading and the size of the colloids. The results are discussed in the subsequent sections.

### 2.1.1 Platinum precursor and surfactant

**Figure 7:**
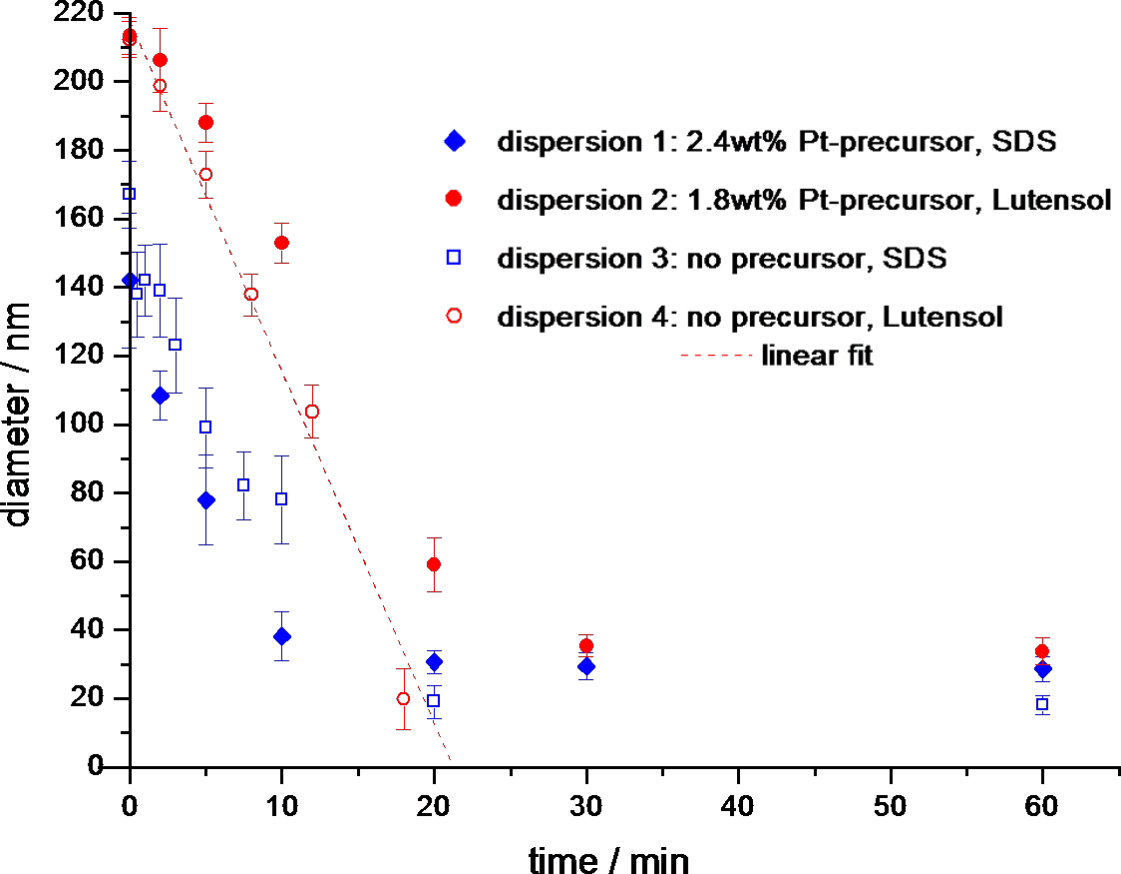
Diameter of Pt-precursor loaded or unloaded PS particles prepared with the surfactants SDS or Lutensol AT50 versus oxygen plasma exposure time. Only particles without Pt-precursor and synthesized with Lutensol AT50 can be removed without residues from the surface (dispersion 4). For all other particles the diameter monotonically decreases and finally approaches a saturation diameter (see also Figure 8).

Based on the observation that the saturated state after etching contains tiny platinum oxide crystallites, it is reasonable to assume that the precursor-complex platinum acetylacetonate has an influence on the saturation. Therefore, colloids were investigated that were prepared by emulsion polymerization with or without the precursor complex, and with SDS or Lutensol AT50 as surfactant. In contrast to SDS, which contains sodium and sulfur, Lutensol AT50 only consists of carbon, oxygen and hydrogen, which are likely to be transformed into volatile products during plasma treatment. The according etching behaviour of the various cases is summarized in Figure 7.

**Figure 8:**
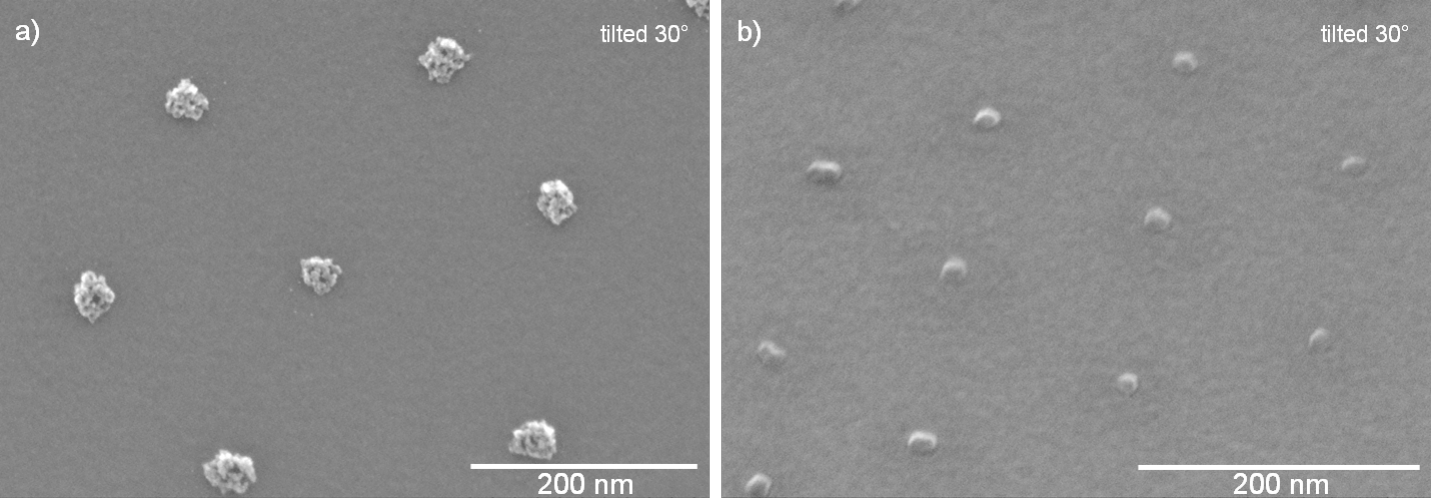
HRSEM images of particles in the saturated state after 60 min isotropic oxygen plasma treatment. a) Loaded with Pt-precursor and synthesized with the surfactant Lutensol AT50 and b) unloaded but synthesized by using SDS (dispersion 2 and 3 of Figure 7).

First, the effect of the surfactant on the plasma assisted size reduction was investigated. For dispersion 1 and 3 the ionic surfactant SDS, and for dispersions 2 and 4 the non-ionic Lutensol AT50, were used. It turned out that for dispersions 1 to 3, containing either Pt precursor or SDS or both, oxygen plasma etching leads to a saturation of the particle diameter (Figure 8). In contrast, colloids of dispersion 4 which did not contain Pt precursor and were synthesized with Lutensol AT50, could be completely removed under identical etching conditions, as proved by HRSEM (Figure 7, linear fit). In summary, the Pt precursor within the colloids as well as the surfactant SDS interferes with the etching process and, thus, leads to the saturated state. As they are not present in dispersion 4, non-volatile components such as metals (Pt, Na) and/or sulfur were responsible for saturation. However, currently one cannot exclude further factors contributing to the appearance of the saturated state.

### 2.1.2 Amount of platinum precursor

In order to analyze the effect of Pt-precursor concentration within the colloids on the saturation diameter after oxygen plasma exposure, a set of three colloidal dispersions, synthesized by surfactant free emulsion polymerization (for details see [12]), were investigated. Here, the loading of the colloids with platinum acetylacetonate was varied from (1 wt %, to 2 wt % and 4 wt %) relative to the monomer while keeping all other parameters constant. After deposition of the initially 175 nm sized colloids onto Si substrates and reducing their size in oxygen plasma, diameters of 27 ± 2, 41 ± 3 and 45 ± 2 nm were found for the corresponding saturated states. Obviously, the total amount of Pt precursor strongly influences the saturation diameter: The higher the precursor content the larger the diameter of the saturated lumps. A simple model may account for this observation. Assuming a homogeneous distribution of Pt precursor within the initial colloids, isotropic etching will reduce the colloidal size by removing volatile species shell-by-shell, whereas the non-volatile constituents such as the Pt will remain on top of the residual particle, which, in this way, will be increasingly protected from further etching. Calculating the total amount of Pt originally present within a shell thickness defined by the difference of the initial radius of the colloid and its value in the saturated state after etching, one arrives at an equivalent Pt layer thickness of around 3 to 4 Å (Pt lattice parameter *a*_Pt_ = 3.92 Å).

A similar result was obtained for Pt-precursor loaded polymethylmethacrylate (PMMA) particles [35]. This suggests that the saturated etching state is obtained whenever the non-volatile Pt produced by the etching approximately forms a protective closed layer around the residual particle. However, the HRSTEM image of Figure 6 does not reveal such a closed film of Pt or PtO_2_ around the lumps. Thus, a more sophisticated model including the role of the SDS component is needed. But the described “monolayer”-result at least delivers a useful empirical rule to predict the size of the saturated particles.

### 2.1.3 Size of the colloids

With the method of the miniemulsion polymerization described in subsection 1, seed particles of 165 ± 14 nm with a loading of 4 wt % Pt precursor (with respect to the monomer) were synthesized in a first step. Subsequently, the particles were enhanced in size by seeded emulsion polymerization, such that they all had identical loading but variable size. Exemplarily, here colloids with diameters of 256 ± 9, 423 ± 19 and 594 ± 48 nm were investigated in detail with respect to their post-etching saturation diameter. The corresponding diameters of the lumps after the plasma treatment were 40, 55 and 97 nm (for details of the size determination see the Experimental section).

**Figure 9:**
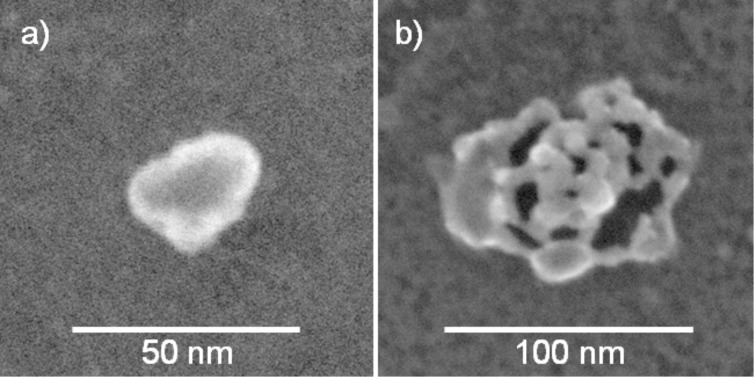
Saturated states after plasma etching: a) Closed surface of a seeded 256 nm particle and b) porous shape of a seeded 594 nm particle, both with a 165 nm precursor-filled core.

It should be noted, however, that the shape of the resulting lumps differs drastically. For the smallest particles, a round, closed shape of the lumps was obtained after the plasma treatment (Figure 9a). The bigger particles exhibited a porous nonspherical shape, as illustrated in Figure 9b. This transition in shape is caused by softening of the colloidal particles during the plasma procedure and, as a consequence, spreading of particle material on the substrate [34]. This leads to a laterally extended lump which is too large to allow agglomeration into a single metal particle during the annealing process. In detail, elements such as sodium and sulfur are removed by annealing, and the platinum oxide is decomposed into platinum as revealed by XPS. Finally, the platinum agglomerates into one NP, sometimes still exhibiting a granular substructure. During the cool-down process in nitrogen the Pt NPs remain stable in the metallic state. The experimental observation that exclusively colloids with initial diameters below 260 nm could be transformed into single NPs by annealing, whereas bigger colloids ended in the granular structure [12], appears to be a serious limit of the colloidal technique.

In summary, the size of the etch-induced lumps is dependent on the initial size of the colloids. A full understanding is hindered by the fact that different initial parameters are interrelated. For example, the initial surface as well as total concentration of surfactant could be different for various colloidal diameters. Furthermore, the contact area of particle and substrate is clearly dependent on the initial size of the colloid. Finally, the distribution of the Pt precursor inside the colloidal particle may also play an important role.

To clarify this last point, TEM and EDX (energy dispersive X-ray spectroscopy) investigations on seed and size enhanced seeded colloids were performed. However, due to the limited element specific sensitivity of these techniques and the small Pt content in the NPs, respectively, it was not possible to resolve any gradients in the distribution of the Pt complex inside the seeded colloids. Nevertheless, it was possible to exclude agglomerates of Pt precursors inside or on the surface of the colloids. It is worth mentioning, that Pt precursor loaded PMMA particles show similar etching-induced saturation behaviour as for loaded PS particles. In both cases, a homogenous diffusion of the Pt precursor during the seeded emulsion polymerization cannot be safely excluded at the moment.

## Conclusion

In this contribution, we applied miniemulsion polymerization to produce metal-complex containing polystyrene particles as a model compound for a functional latex particle. A seeded emulsion polymerization process to increase the size and monodispersity of the particles was investigated. Finally, as an application of the newly developed process, the preparation of platinum nanoparticle arrays was demonstrated exhibiting high homogeneity and lateral order. For this purpose, a plasma-assisted removal of the organic material of the colloids was applied.

The following conclusions regarding experimental conditions of the seeded emulsion polymerization reactions and Pt-particle generation processes can be drawn from the experiments performed.

1) It is possible to synthesize functional colloidal particles with a diameter up to 600 nm in one step. Larger diameters have to be synthesized in a step-by-step fashion.

2) The initiator concentration has to be high in order to induce polymerization and not only depends on the amount of monomer added, but also on the amount of the continuous phase. The best results were achieved using 10–25 wt % APS relative to the added monomer.

3) The choice and concentration of the surfactant is critical for a successful reaction. SDS as an anionic surfactant proved to be superior to Lutensol AT50 as non-ionic surfactant. High surfactant concentrations lead to more homogeneous particles but, eventually, favour secondary nucleation. Therefore, the surfactant concentration ought to be as small as possible in order to stabilize the dispersion without inducing secondary nucleation.

4) The composition of the continuous phase also influences the reaction. Small amounts of added salt lead to final particle sizes reaching the theoretical diameter, whereas higher salt amounts deteriorate the stability of the dispersion. Ethanol may be added to improve the monodispersivity of the samples. However, care has to be taken as secondary nucleation seems to be favoured as well.

5) The precursor platinum acetylacetonate inside the PS colloids and the surfactant SDS causes saturation of the particle diameter during the oxygen plasma induced etching.

6) The diameter of the particles in the saturated state depends on the total amount of Pt precursor inside the PS particles as well as on the initial size of the colloids.

7) The interparticle distance is presently limited to about 260 nm.

## Experimental

### Miniemulsion polymerization

Hexadecane (250 mg), 2,2'-Azobis(2-methylbutyronitril) (V59, 100 mg) and platinum(II) acetylacetonate (60 mg) were dissolved in monomer (6 g) under continuous stirring. To this phase, a mixture of water (milliQ quality) and SDS (60 mg) was added. After stirring for one hour at 1800 rpm and at room temperature, miniemulsification was achieved by ultrasonication of the mixture under ice-cooling for 120 s with a 1/2” tip at 90% amplitude, following a 10 s pulse-10 s break-protocol (Branson digital sonifier 450-D, Dietzenbach, Germany). Subsequently, the mixture was heated to 72 °C and polymerized for 12 h under gentle continuous stirring. After cooling to room temperature, the colloidal dispersion was filtered and extensively dialysed (Visking tubes, MWCO 14.000 g/mol, Carl Roth, Karlsruhe, Germany) until the conductivity of the water phase after dialysis was similar to that for deionized water.

#### Seeded polymerization

The protocol given here can be considered the standard protocol. All reactions deviating from this protocol are specified in the main text. A dispersion of 0.1 wt % of seed particles with 0.01 wt % of SDS (relative to the amount of water used) was heated to 75 °C. Ammonium peroxodisulfate, (APS, (NH_4_)_2_S_2_O_8_), and 10–15% of the monomer added was dissolved in a small amount of ultra-pure water and added to the dispersion. Styrene as monomer was added to the solution using a syringe pump with a flow rate of 1 mL·h^−1^. The amount of monomer was varied to obtain different sizes of the resulting seeded particles. The reaction was stirred for 24 h at 80 °C under an argon atmosphere. After completion, the final dispersion was dialysed extensively.

#### Plasma assisted combustion of the colloidal particles to produce nanoparticle arrays

The plasma was delivered by a commercially available etching machine (Oxford Plasmalab 80 Plus RIE) with an inductively coupled plasma source (ICP). The colloids were etched by an isotropic oxygen plasma [11–12].

#### Annealing

After finishing the plasma treatment, the particles were annealed at 1100 °C in a commercially available lamp furnace (UniTemp RTP-1200-100) in an oxygen atmosphere at 1 mbar for 10 min and cooled down in a nitrogen gas-flow to RT.

#### Sample Characterization

Scanning electron microscope images of the colloidal particles were recorded on a Gemini 1530 microscope (Carl Zeiss AG, Oberkochen, Germany).

High resolution SEM images were taken on a Hitachi S5200 with an acceleration voltage of 30 kV guaranteeing a resolution of 0.5 nm.

TEM was carried out using a FEI Titan 80-300 (FEI, Eindhoven, Netherlands) operating at 300 kV in the scanning mode (STEM). The images were acquired using a mass sensitive high annular dark-field detector (HAADF, type Fischione) resulting in a resolution of < 0.135 nm.

Images were evaluated by the use of the program ImageJ. The diameters of the particles in the saturation state were determined as follows: First the outer rim of the particles was defined, then it was assumed that the total area inside this rim equals the projected area of a round particle, and hence the diameter was calculated.
